# Conditional ablation of the RFX4 isoform 1 transcription factor: Allele dosage effects on brain phenotype

**DOI:** 10.1371/journal.pone.0190561

**Published:** 2018-01-03

**Authors:** Ping Xu, James P. Morrison, Julie F. Foley, Deborah J. Stumpo, Toni Ward, Darryl C. Zeldin, Perry J. Blackshear

**Affiliations:** 1 Signal Transduction Laboratory, National Institute of Environmental Health Sciences, Research Triangle Park, North Carolina, Unites States of America; 2 Pathology, Charles River Laboratories, Shrewsbury, Massachusetts, Unites States of America; 3 Cellular and Molecular Pathology Branch, National Institute of Environmental Health Sciences, Research Triangle Park, North Carolina, Unites States of America; 4 Immunity, Inflammation and Disease Laboratory, National Institute of Environmental Health Sciences, Research Triangle Park, North Carolina, Unites States of America; Texas A&M University, UNITED STATES

## Abstract

Regulatory factor X4 (RFX4) isoform 1 is a recently discovered isoform of the winged helix transcription factor RFX4, which can bind to X-box consensus sequences that are enriched in the promoters of cilia-related genes. Early insertional mutagenesis studies in mice first identified this isoform, and demonstrated that it was crucial for mouse brain development. RFX4 isoform 1 is the only RFX4 isoform significantly expressed in the mouse fetal and adult brain. In this study, we evaluated conditional knock-out (KO) mice in which one or two floxed alleles of *Rfx4* were deleted early in development through the use of a *Sox2*-Cre transgene. Heterozygous deletion of *Rfx4* resulted in severe, non-communicating congenital hydrocephalus associated with hypoplasia of the subcommissural organ. Homozygous deletion of *Rfx4* resulted in formation of a single ventricle in the forebrain, and severe dorsoventral patterning defects in the telencephalon and midbrain at embryonic day 12.5, a collection of phenotypes that resembled human holoprosencephaly. No anatomical abnormalities were noted outside the brain in either case. At the molecular level, transcripts encoded by the cilia-related gene *Foxj1* were significantly decreased, and *Foxj1* was identified as a direct gene target of RFX4 isoform 1. The phenotypes were similar to those observed in the previous *Rfx4* insertional mutagenesis studies. Thus, we provide a novel conditional KO animal model in which to investigate the downstream genes directly and/or indirectly regulated by RFX4 isoform 1. This model could provide new insights into the pathogenesis of obstructive hydrocephalus and holoprosencephaly in humans, both relatively common and disabling birth defects.

## Introduction

Hydrocephalus, excessive accumulation of fluid in the brain, is a common birth defect, with the prevalence of congenital hydrocephalus in the United States and Europe between 0.5 and 0.8 per 1000 births [1]. Hydrocephalus may result from inherited genetic abnormalities or developmental disorders such as folic acid deficiency. To date, only four gene mutations have been identified in patients with severe congenital hydrocephalus, mapping to *MPDZ* [2], *L1CAM* [3], *AP1S2* [4], and *CCDC88C* [5]. More than a hundred genes have been implicated in different models of rodent hydrocephalus [6]; however, the genetic causes of hydrocephalus are far from understood in either mice or humans.

Regulatory factor X (RFX) proteins are helix-turn-helix transcription factors, and are encoded by seven *RFX* genes *(1–7)* in humans [7, 8]. *Rfx* genes have been found in many eukaryotic species, including yeast, fruit flies, mice and humans [7, 8]. Aberrations in *Rfx3* and *Rfx4* have been linked to hydrocephalus in mice [9, 10]. RFX family proteins share a conserved DNA binding domain, and bind to “X-box” consensus sequences in the promoter regions of target genes, an observation first noted in MHC class II gene promoters [11].

We first identified the transcript encoding what is now known as RFX4 isoform 1 in mice expressing a transgene encoding a cardiac-specific cytochrome P450 epoxygenase that developed hydrocephalus; we demonstrated that the transgene had disrupted the *Rfx4* gene, leading to the absence of RFX4 isoform 1 expression [10]. In current nomenclature, mouse RFX4 isoform 1 (GenBank accession number NP_001020089) is encoded by *Rfx4* transcript variant 1 (NM_001024918); this was referred to as *RFX4* transcript variant 3 in our original publication [10]. Mouse RFX4 isoform 1 is orthologous to human RFX4 isoform c (NP_998759), and the two proteins are 97% identical. The transcript encoding RFX4 isoform 1 is the only *Rfx4* gene product significantly expressed in the mouse brain, spinal cord, and eye (our unpublished data).

In our earlier study, heterozygous insertional mutant mice developed obstructive hydrocephalus with severe hypoplasia of the subcommissural organ (SCO), whereas homozygous insertional mutant mice exhibited more severe brain malformations [10] and death in the early perinatal period. To begin to address the contributions of *Rfx4* in specific cell types, we generated mice with a floxed *Rfx4* allele that removed the DNA binding domain encoded by exon 4. In these initial experiments, we bred these mice to *Sox2*-Cre mice, so that Cre-recombinase would be expressed in epiblast cells after embryonic day (E) 6.5, effectively driving Cre-recombinase activity in the whole organism during development [12]. We found that mice heterozygous for this *Rfx4* deletion developed congenital hydrocephalus, whereas the mice with the homozygous deletion in E12.5 embryos exhibited a condition that resembles human holoprosencephaly, a disease in which the forebrain of the embryo fails to develop into two hemispheres. Our data suggest that this may be due, at least in part, to the dysregulation of RFX4 isoform 1-regulated expression of the cilia-related gene *Foxj1*.

## Methods

### Generation of floxed *Rfx4* mice and breeding with *Sox2-*Cre mice

**Fig 1:**
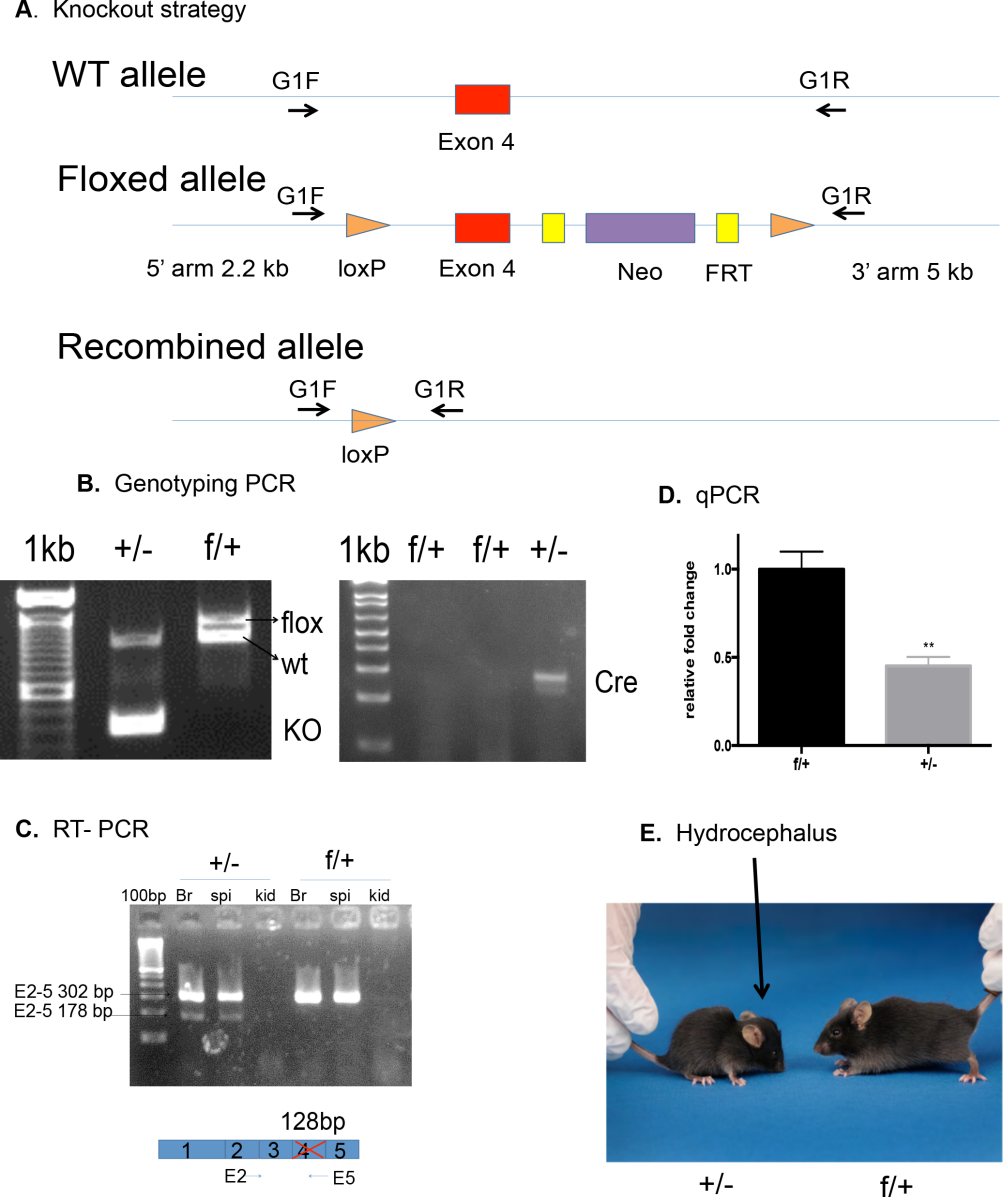
Conditional knockout and genotyping strategies. (A) Schematic version of the strategy used for the generation of the floxed *Rfx4* KO mice. In the WT allele, exon 4 was chosen for deletion. In the floxed allele, two loxP sites flanked exon 4, and a neomycin (neo) cassette with two Frt sites was inserted. Cre recombinase causes deletion of exon 4. “G1F”, on the 5’ arm, and “G1R”, on the 3’ arm, are genotyping PCR primers which produce WT, flox or KO bands. (B) Genotyping PCR: Lane +/- refers to a *Rfx4****^+/-^*** mouse, and the two bands were from floxed and KO alleles from a KO mouse; lane f/+ refers to a control mouse, and the two bands were from floxed and WT alleles from a *Rfx4****^f/+^***mouse. (C) RT-PCR demonstrated a lower band in *Rfx4****^+/-^*** brain and spinal cord, representing the transcript produced by the KO allele. (D) As determined by qPCR, *Rfx4* mRNA levels were decreased by approximately 50% (P< 0.01, n = 4) in the brains of *Rfx4****^+/-^*** mice, as determined by fold changes after normalizing with *Gapdh* mRNA. (E) Image of the domed head of a mouse at 4 weeks of age (left) that was heterozygous for the *Rfx4* KO.

Heterozygous mice with a floxed *Rfx4* allele were generated by gene targeting in C57BL/6 embryonic stem (ES) cells. Since it contains the DNA binding domain, exon 4 (0.9 kb) of *Rfx4* (bases 615–738 of GenBank accession number NM_001024918) was chosen for the floxed KO region, and was flanked by loxP sites; the vector included a 2.2 kb 5’ homology arm and a 5.0 kb 3’ homology arm. For positive ES cell selection, the Neo expression cassette flanked by FRT sequences (for the subsequent removal of the Neo cassette) (Fig 1A), and a diptheria toxin-A gene fragment (DTA) expression cassette (for negative selection of the ES cells), were cloned into LoxFtNwCD vectors. Flp sites were removed before injection into blastocysts. Heterozygous mice were obtained by breeding the chimeras with C57BL/6Tac wild type (WT) females. The mice were generated by Caliper Discovery Alliances & Services Company (Hanover, MD). *Sox2*-Cre mice on a C57BL/6 background were purchased from Jackson Laboratory (Stock 008454, Bar Harbor, Maine), and *Rfx4^flox/flox^* mice were bred with the *Sox2*- cre mice. *Rfx4*^+/-^ mice were generated when Cre recombinase was transmitted [13]. In these mice, Cre- recombinase driven by the *Sox2* promoter should be expressed in embryonic tissues but not in extraembryonic tissues [12, 14].

Animals were genotyped at 3–4 weeks of age by performing PCR on genomic DNA prepared from tail biopsies. The genotyping primers were targeted to the flanking site as GO1F sequence 5'- AGTATTTTGTTCCCCTTTCT-3’ and GO1R sequence 5'-TTATAACGGTGTGAGGGTT-3'. Cre- recombinase primers were used as follows: Cre1 forward 5'- GGACATGTTCAGGGATCGCCAGGCG-3' and reverse 5'- GCATAACCAGTGAAACAGCATTGCTG-3'.

### Creation of homozygous *Rfx4* KO Mice

To generate *Rfx4* KO embryos, male heterozygous mice (flox/wt (+) Cre positive (+)) were bred to flox/+ or +/- mice to generate homozygous floxed mice (flox/flox) that were Cre-positive or Cre-negative (controls). Pregnant females were euthanized with CO2 prior to embryo removal at specified times during gestation, and embryos were harvested. All studies were approved by the NIEHS Animal Care and Use Committee.

### Perfusion and fixation of the adult brains, histology, and immunostaining

For collection of brains from adult mice, age-matched KO and control mice were perfused under anesthesia using 50 mg/kg sodium pentobarbital. Briefly, a needle was inserted into the left ventricle, and the right atrium was cut to provide drainage. A steady flow of approximately 20 ml/min of 0.9% saline solution with 10 U/ml heparin (Sigma H0777) was maintained until the perfusate was clear. The mice were then perfused with a 4% paraformaldehyde solution until fixation was achieved.

For routine histology, embryos and tissues from neonatal or adult mice were immersion fixed in 4% paraformaldehyde for 24–48 hours, depending on the tissue size. They were then embedded in paraffin, sectioned into 5–8 μm sections and stained with hematoxylin and eosin (H&E) by standard methods.

For immunostaining, sections were incubated in 3% H2O2 to inactivate endogenous peroxidases, followed by antigen retrieval with heat and pressure in citrate buffer (Biocare Medical, Concord, CA). Endogenous biotin was blocked with Avidin-Biotin blocking reagents (Vector Laboratories, Burlingame, CA). Sections were then incubated with Reissner's fiber [15] antibody (1:1000) [16] (a generous gift from Dr. E. M. Rodriguez, Instituto de Histologia Patologia, Universidad Austral de Chile, Valdivia, Chile). Consecutive sections (5 μm/section) from the whole brain were stained, and the length of SCO from anterior to posterior was considered to be five times the number of positive sections. Ki67 staining used anti-Ki67 antibody (1:500, ab16667, Abcam, Cambridge, United Kingdom) for 60 min, followed by peroxidase-conjugated streptavidin labeling (Biogenex Laboratories, San Ramon, CA) for 30 min. Immunolabeled antigen-antibody complexes were visualized using diaminobenzidine. The sections were lightly counterstained with hematoxylin before analysis. Cilia were stained with acetylated-alpha tubulin antibody (1:200, T7451, Sigma Aldrich, St. Louis, MO), followed by Alexa Fluor 594 anti-mouse antibody (1:1000, Invitrogen, Carlsbad, CA, USA) incubation for 1 hour, counterstained with DAPI, and fluorescent images were taken with a Zeiss 710 microscope (Carl Zeiss AG, Oberkochen, Germany).

### Total RNA extraction and quantitative real time PCR (qPCR)

Total brain RNA was extracted using Trizol (Invitrogen, Carlsbad, CA, USA), and was reverse- transcribed into first-strand cDNA using the SuperScript® III First-Strand Synthesis System (Invitrogen, Carlsbad, CA, USA) according to the manufacturer’s instructions. Real-time RT-PCR reactions used the SYBR master mix (Applied System, Foster City, CA), and the amplifications were performed as follows: 2 min at 50°C, 10 min at 95°C, then 40 cycles each at 95°C for 15 s and 60°C for 60 s in the ABI/Prism 7900 HT Sequence Detector System. The primer sets used are listed 5' to 3', with the forward primer listed first. Results were normalized to glyceraldehyde-3-phosphate dehydrogenase (*Gapdh*) mRNA as a reference control transcript, and calculated using the relative quantification method 2^ΔΔCq^ [17]. Primer sequences were:

RT, GATGTCTCAATGAAAGCGAG and CCCGAGTCTTCTGGTGG;

*Rfx4* (flanking exon 4–5), TGTCAATGCTGCCAGCTTT and CCCGAGTCTTCTGGTGGTTA;

*Gapdh*, GCACAGTCAAGGCCGAGAAT and GCCTTCTCCATGGTGGTGAA;

*Foxj1*, GCTACTTCCGCCATGCAGAC and CTTCTCCCGAGGCACTTGA;

*Rfx3*, TCCGACCCAGTCTCTGATGT and AAGGCCACTTGGTCATTCTG;

*Rfx2*, CTCGGAATTCTGCATGCTC and TTACTTACCAACGGGGACCA;

*Dync2li1*, GGTGAGCCGGAATACAGAGAA and TGTTTGGTAGGATCTGGGACA;

*Thbn*, GAAGCAACAAGTGGTGTCAGT and ACAGTCTATGTAGAGTTGAGCCC.

### Plasmids

The full-length mouse *Rfx4_* isoform 1cDNA was amplified by PCR using the following primers: 5′- aaatttGGTACCAAGAGCATGCATTGTGGGTTACTG-3′ and 5′- aaattCTCGAGTCACTTGTCATCGTCGTCCTTGTAATCCTTAGCCCATCCAGTGGAGGCCTC-3′. PCR products were gel-purified, digested with Asp781 and XhoI (NEB, Ipswich, MA) and ligated into the vector CMV-Flag-BGH3’/BS+ cut with the same enzymes [18].

To clone the 5′-upstream region containing the putative X-box of the mouse *Foxj1* gene, PCR was carried out with mouse genomic DNA using the forward primer 5′- ATCTGAGCTCAAGGGCACGGTTCCCCG -3′ (corresponding to bases 19821580–92 of GenBank accession number NT_078575.2) and the reverse primer 5′- TCAATAAGATCTGACGCTCGGAAGGCTTCTTCT -3′ (bases 19822565–77). The 5′-upstream region outside of the X-box fragment was amplified by using the forward primer 5′- AGTAATGAGCTCTGGGGAACTAGTCCTGTC-3’, and the reverse primer 5′- GAACCCCAAAGCTGATGGCAACTCGAGTATT-3’. All of the PCR products were digested with SacI and XhoI, purified, and then cloned into the pGL4.23 promoter vector (Promega, Madison, WI). All constructs were confirmed by sequencing.

### Chromatin immunoprecipitation (ChIP) assay

ChIP experiments were performed using the ChIP Assay Kit (Millipore, Billerica, Massachusetts, USA) according to the manufacturer's instructions. Adult brains were cut into pieces and fixed in 1% formaldehyde, and DNA was isolated and sheared into 500–1000 bp fragments using a Bioruptor TM sonicator (Diagenode, NY, USA). Sheared chromatin was pre-cleared with protein G beads (sc-2027, Santa Cruz, Dallas, Texas), and 10 μl of supernatant was saved as ‘input DNA’. Half of the remaining supernatant was incubated with 2 μg rabbit serum IgG (sc-2027, Santa Cruz, Dallas, Texas) at 4°C overnight, and another half of the supernatant was incubated with 2 μg of anti-RFX4 polyclonal antibody (Aviva Systems Biology, San Diego, CA). The chromatin-antibody mixture was then incubated with protein G beads for 1 hour at 4°C. After the reverse cross-linking step, immunoprecipitated DNA was treated with RNase A and proteinase K and eluted from the beads, then purified from the mini-columns. The eluted DNA was amplified with the primers for the *Foxj1* promoter: X-box2, 5’-TGAGGGCAAAGACTTCAAGG-3’ and 5’-GATCCGACTCTGTGCATTCC-3’; X-box3, 5’-AGAGAGTTGCCGCCAGAGG-3’ and 5’-AGACGCTCGGAAGGCTTCTT-3’.

### Transient transfection and luciferase reporter assays

The mouse neuroblastoma/rat glioma hybrid cell line NG108-15 (American Type Culture Collection, Manassas, VA, USA) was maintained in Dulbecco's modified Eagle's medium supplemented with 10% (v/v) fetal bovine serum and 100 units/ml penicillin/streptomycin. For reporter assays, cells were grown in 24-well plates to 60% confluence, and then transiently co-transfected with the *Rfx4* cDNA expression vector or the control vector (0.1 μg), along with a luciferase reporter containing the *Foxj1* promoter that included putative X-box sequences in the pGL4.23 vector (0.3 μg), using Lipofectimine 2000 reagent (Invitrogen, Carlsbad, CA, USA) for transfection. Plasmid pRL-CMV40 (5 ng) was also co-transfected to normalize transfection efficiency. Transfection assays were performed in duplicate. Eighteen hours after transfection, the cells were washed once with phosphate-buffered saline, and lysed in Passive Lysis Buffer (provided by the Dual-Luciferase Reporter Assay Systems (Promega, Madison, WI). Luciferase activities in the lysates were measured by the TECAN infinite 2000 (Tecan, Männedorf, Switzerland), according to the manufacturer's protocol.

### Statistical analysis

All the statistical analyses were performed using GraphPad Prism 6.02 (GraphPad Software, San Diego California). All data are expressed as mean +/- SEM; a p-value <0.05 was considered statistically significant. * p<0.05, ** p<0.01, *** p<0.001, **** p<0.0001.

## Results

### Generation of *Rfx4* KO mice

For this study, we chose exon 4 of *Rfx4*, encoding the DNA binding domain of the RFX4 isoform 1 protein, to be floxed (Fig 1A). This contrasts with our previous model, which involved a random insertion that interrupted the intron between exon 17 and 18 of *Rfx4* [10]. In the current study, heterozygous and homozygous floxed *Rfx4* mice were healthy and fertile. The genotypes of the mice were determined by PCR, using primers that flanked the *Rfx4* exon 4 sequences. The largest band of 1420 bp (Fig 1B) can be attributed to the floxed allele, the middle band of 1380 bp to the WT allele, and the lowest band of approximately 300 bp to the KO allele. A separate 300 bp PCR band was used as a positive control for the Cre-recombinase genotype, using a generic Cre primer pair (Fig 1B).

In order to confirm that the Cre-recombinase produced the desired deletion in transcripts expressed in the brain, primers flanking exons 2 to 5, as reflected in the *Rfx4* mRNA, were used to detect transcript differences between the WT and KO alleles. A 178 bp band was detected by RT-PCR only in the *Rfx4*^+/-^ mice, in contrast to the 302 bp band from the control mice (Fig 1C). Moreover, the levels of mutant transcripts in the brain of *Rfx4^+/-^* mice were decreased by approximately 50% when compared to the control mice, as quantified by qPCR (p<0.01) (Fig 1D). Sequencing the gel-extracted PCR band from the heterozygous mice revealed the complete deletion of exon 4 sequences.

### Hydrocephalus, dysplasia of the SCO and patchy motile cilia in *Rfx4*^+/-^ mice

**Fig 2:**
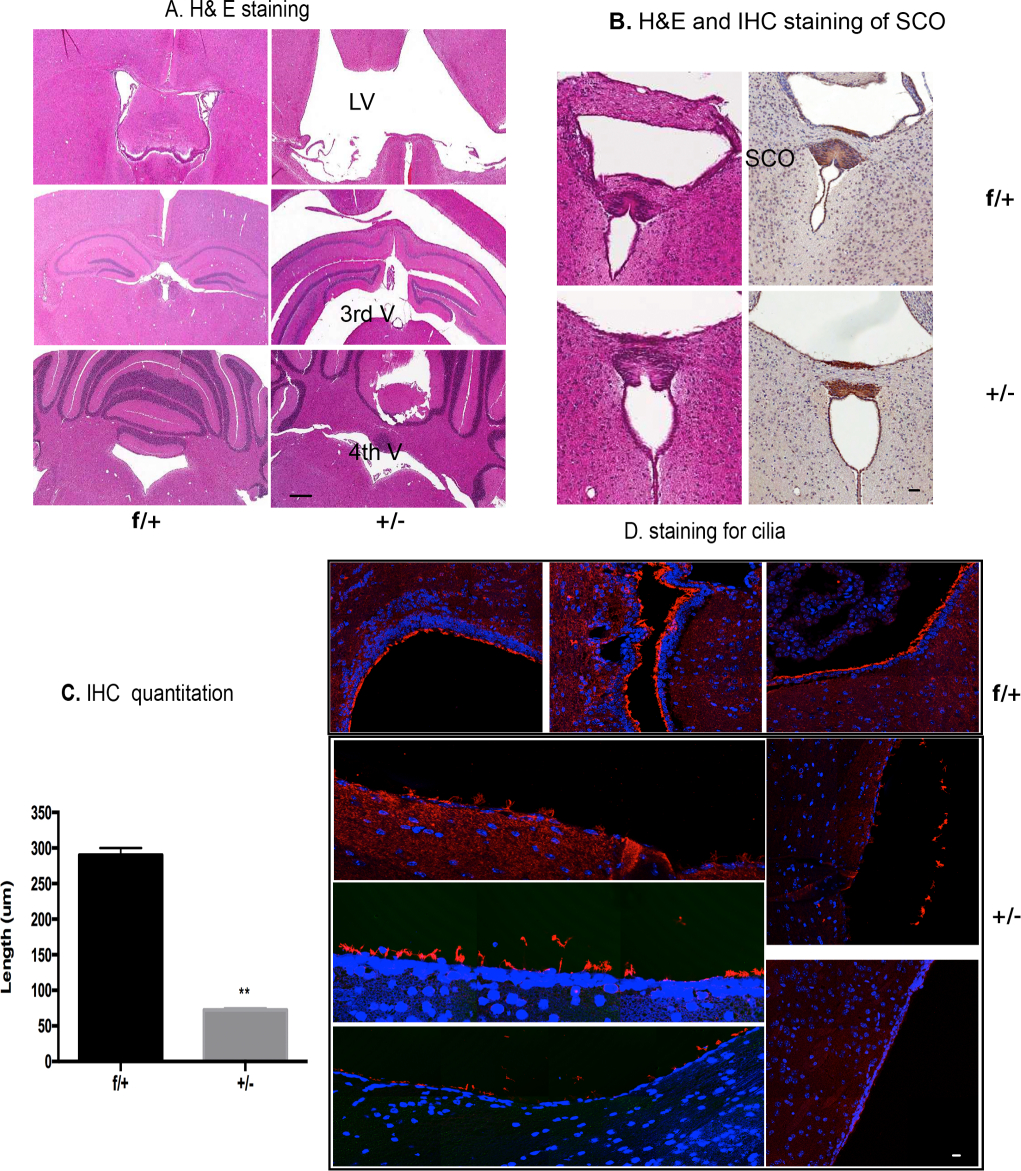
Characterization of *Rfx4^+/-^* mice with hydrocephalus. (A) H&E staining. The left panels are representative slides from a control mouse at 4 weeks of age (*Rfx*^***f/+***^), and the right panels were from a *Rfx4****^+/-^*** mouse, with roughly equivalent sections shown descending from rostral to caudal. The sections demonstrate the enlarged lateral and third ventricles, as indicated by the arrows, but not fourth ventricle enlargement, in the *Rfx4*^+/-^ mouse (representative of 6 *Rfx4*^^+/-^^ mice and 4 controls). The middle right panel shows the emergence of false ventricles, and the white matter appeared to be compressed from the severe hydrocephalus. Scale bar = 20 μm. (B) SCO hypoplasia in *Rfx4*^+/-^ mice. The left panels of B showed the SCO stained with H&E, with a section from a control mouse (*Rfx*^*f/+*^) on top, and from an *Rfx4*^+/-^ KO mouse on the bottom. Neighboring sections were stained with an antibody to Reissner’s fibers, and the resulting immunohistochemistry is shown on the right. Scale bar = 10 μm. (C) IHC quantitation. SCO length was measured by counting positively stained Reissner’s fibers slides from 3 controls and 3 *Rfx4*^+/-^ mice, using adjacent sections cut 5 μm apart. These measurements showed that SCO length in *Rfx4*^+/-^ KO mice was decreased when compared to controls (p<0.01). (D) Immunofluorescence staining. Acetylated alpha-tubulin labeled cilia (red fluorescence, blue is DAPI) were detached, patchy or missing from the walls of the ventricles in *Rfx4*^+/-^ mice (lower five panels). Scale bar = 5 μm.

Within the first 8 weeks of life, almost half of the heterozygous mice (83 of 186) developed domed heads, an external sign of hydrocephalus (Fig 1E). This appeared as early as 3 weeks of age. By 4 weeks, 24% of mice had developed domed heads, an additional 19% occurred by 8 weeks, and only 3 additional mice developed domed heads after 8 weeks. There was no difference in the incidence of hydrocephalus occurrence between the males and females. When hydrocephalus was present in mice at 4 weeks of age, enlarged lateral and third ventricles were present, along with large ‘false’ ventricles in the cerebral hemispheres between the caudate, putamen and the cerebral cortex, as shown in H&E stained sections (Fig 2A). Small bundles of stretched white matter often spanned the false ventricles, and there was considerable atrophy of the cerebral cortex and compression of the brainstem. The ependyma was partially absent over a large portion of the ventricular surface. The mesencephalic aqueduct and fourth ventricle appeared normal. This phenotype resembled, but was less severe than, that of heterozygous mice in the *Rfx4*-insertional mutagenesis model [10], in which there was a 100% frequency of congenital hydrocephalus that was also characterized by enlarged olfactory ventricles, not seen in the current floxed KO model.

Two main types of hydrocephalus can be defined by the location of cerebrospinal fluid (CSF) accumulation in the brain. Communicating hydrocephalus (non-obstructive hydrocephalus) is caused by inadequate absorption of CSF by the subarachnoid cisternae, whereas non-communicating hydrocephalus–also called "obstructive" hydrocephalus–occurs when the flow of CSF is blocked at the aqueduct, with CSF accumulating in the ventricles. Since no excess CSF was present in the subarachnoid spaces of these *Rfx4^+/-^* mice, this represents a model of non-communicating hydrocephalus, as was true in the insertional mutation model.

The subcommissural organ (SCO), an ependymal gland located in the dorsocaudal region of the third ventricle at the entrance of the Sylvian aqueduct, a structure that secretes Reissner’s fibers, has been shown to be critical for the patency of the aqueduct [19], and malformations of the SCO can cause hydrocephalus in rodents [20]. In the developing SCO, *Rfx4* transcripts are strongly expressed from E12.5 to birth [10]. Using an antibody specific to Reissner's fibers, we detected SCOs (Fig 2B) that were approximately 300 μm long on average (as seen in coronal serial sections) in the control mice, but they were only approximately 75 μm long on average in the *Rfx4^+/-^* mice with hydrocephalus (Fig 2C) (P< 0.01, n = 3). Thus, the SCO was hypoplastic in the *Rfx4^+/-^* adult mice with hydrocephalus. This lesion was also less severe than that seen in the *Rfx4*-insertional model, in which the SCO was barely detectable or undetectable. Interestingly, it has previously been observed that mutated *Rfx3* hydrocephalic mice also exhibited SCO agenesis, as well as ependymal cell differentiation defects [9].

The cerebral ventricles are lined by ependymal cells, a cell type that has motile cilia, thought to facilitate CSF circulation. We labeled these motile cilia with antibodies to acetylated-alpha tubulin (a marker for cilia) [21]. In the control mice, cilia were present in cells lining the lateral and third ventricles (Fig 2D, upper 3 images), but, in the *Rfx4^+/-^* mice with hydrocephalus, cilia were apparently detached in some areas from the cells lining the walls of the ventricles, or they were patchy or missing in some areas (Fig 2D, lower 5 images). Neither the cilia length, nor their thickness (as measured by the stacking images), were different from those of the control mice (data not shown).

### A dorsal-ventral patterning defect in homozygous *Rfx4^-/-^* mice at E12.5 resembles that of human holoprosencephaly

The breeding of heterozygous *Rfx4^+/-^* mice resulted in only two live homozygous *Rfx4*^-/-^ pups out of 38 born dead (5%), suggesting that homozygosity was largely embryonic lethal. At E16.5, there were two apparently viable homozygotes out of 36 embryos (5.5%), and three out of 25 apparently viable embryos at E14.5 (12%). These values are well below the expected frequency of 25%, suggesting that *Rfx4*^-/-^ embryos had difficulty surviving to E14.5 and beyond.

**Fig 3:**
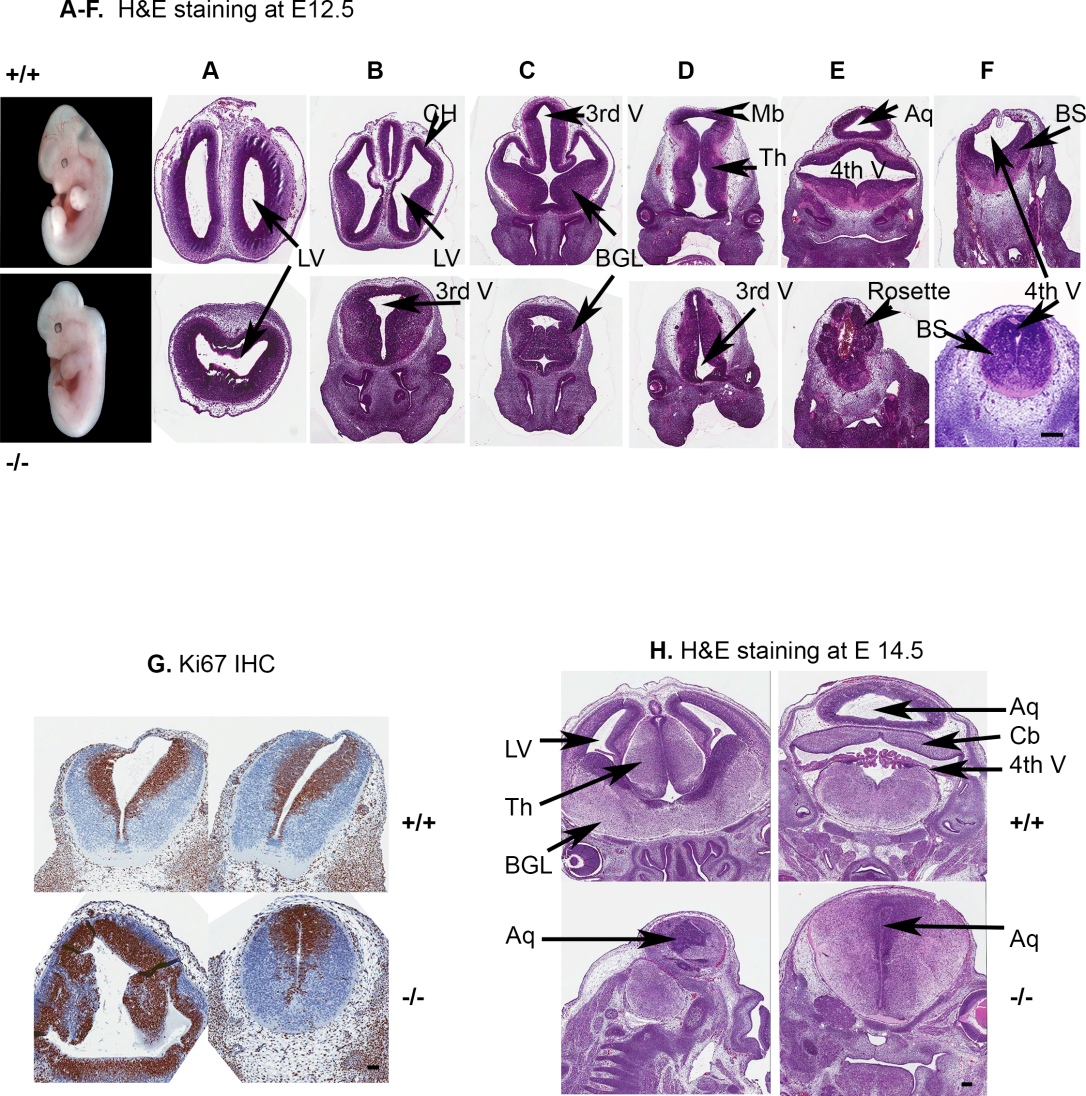
*Rfx4*^-/-^ embryos at E12.5 and E14.5 exhibit holoprosencephaly. Note the complete absence of the developing cerebral hemispheres (CH) in the KO embryo seen in A-C. There was a striking decrease in size of all regions of the brain, but especially the developing midbrain in (D). In addition, the developing mesencephalic aqueduct was absent, and marked neuroepithelial dysplasia was present in the caudal brainstem in (E), characterized by generalized disorganized growth and numerous rosettes. The 4^th^ ventricle was nearly absent in the KO embryo seen in (E-F). Scale bar = 20 μm. (G) The neuroepithelial cells of embryos at E 12.5 were labeled with the Ki67 antibody; the KO embryos (lower right panels) showed less staining overall. However, rosettes representing underdeveloped sites were extensively stained. Scale bar = 10 μm. (H) In representative staining of the brain sections with H&E at E14.5, there was a single ventricle without cerebral hemispheres in the forebrain (lower left panels). LV, lateral ventricle; BG, basal ganglia; Mb, midbrain; Th, thalamus; MA, mesencephalic aqueduct; 3^rd^ V, 3^rd^ ventricle; 4^th^ V, 4^th^ ventricle; BS, brain stem. Scale bar = 10 μm.

Our previous studies using the homozygous *Rfx4* insertional mutation embryos showed that a single ventricle was present in the forebrain at E12.5 [10]. In the present experiments, the *Rfx4*^-/-^ embryos exhibited severe changes in the developing central nervous system. The following pathological description is based on an analysis of KO embryos at E12.5 days of age, and represents the most severe phenotypes observed, as well as the common phenotype. The volume of the vesicles was moderately decreased, as was the size of the entire developing brain (Fig 3A–3F, lower panel). This embryo exhibited extensive hypoplasia throughout the developing brain. From anterior to posterior, in the most severe embryos, there was no histologic evidence of the telencephalic vesicles (developing cerebral hemispheres) in any section (cerebral aplasia). In addition, dorsal structures were fused and lacked an obvious midline (Fig 3A and 3B), a defect that can be observed in the E14.5 sections (Fig 3H). In contrast, ventral structures were better separated (Fig 3C). This striking alteration represents a dorsoventral patterning defect. Normally, this process develops in such a way that the telencephalon becomes specified into dorsal (pallial) and ventral (subpallial) regions. These defects were similar to those seen in our previous insertional mutation model, as well as in the point mutation *Rfx4 ^L298P^* model generated with ENU mutagenesis [22]. This forebrain defect partially mimics human holoprosencephaly, where there is incomplete separation of the anterior portion of the forebrain. It is more similar to semilobar holoprosencephaly, in which the left and right frontal and parietal lobes are fused and the interhemispheric fissure is only present posteriorly. The diencephalon was hypoplastic, but the cellular organization appeared fairly normal (Fig 3D). The developing cerebellum was markedly dysplastic, and contained numerous neuroepithelial rosettes and a lack of overall organization (Fig 3E), also shown by extensive staining with antibodies to the proliferation marker Ki67 (Fig 3G). These rosettes suggested hyperproliferative but underdeveloped cells compare to the littermate controls. Overall, Ki67 stained with less intensity in the mutant mice than the control (Fig 3G, lower right). The roof plate of the fourth ventricle was absent and the neuroepithelium was moderately dysplastic in the caudal brainstem (Fig 3F) and cranial spinal cord.

### *Foxj1* is *trans*-activated by RFX4 isoform 1 in NG108-15 cells

**Fig 4:**
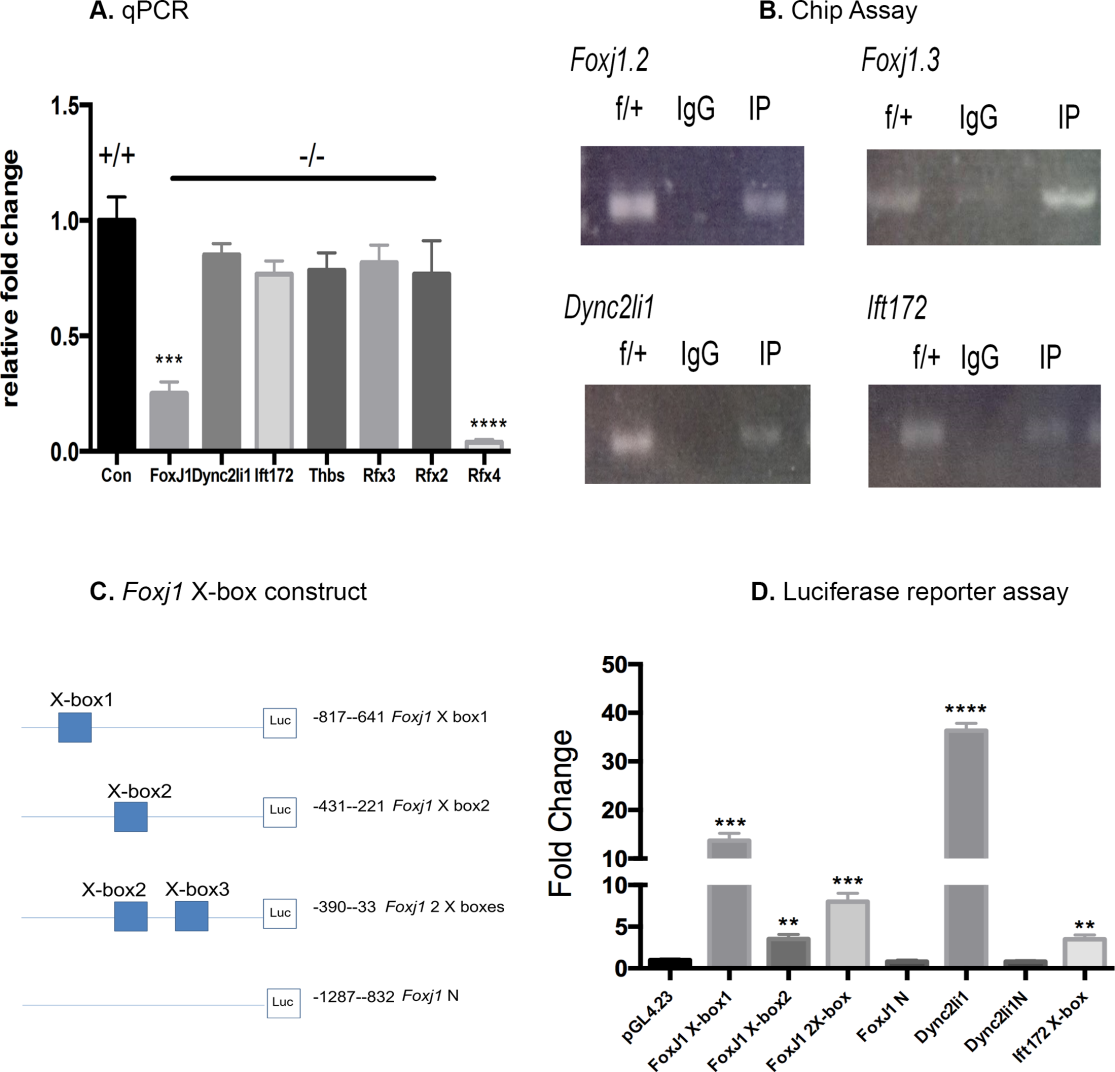
*Foxj1* as a target gene for RFX4. (A) qPCR. *Foxj1* mRNA levels were decreased in E12.5 KO brains, whereas there were no significant differences in *Ift172*, *Thbs*, *Dync2li1*, *Rfx2*, *Rfx3* mRNAs, and almost undetectable *Rfx4* transcripts compared to the controls (P< 0.0001, n = 5). (B) ChIP Assay. Fragmented DNA from adult brain was enriched by RFX4 antibody in the *Foxj1* promoter; the PCR bands shown were from flanking X-box1 and X-box2 primers (upper panel). In the *Dync2li1* and *Ift 172* promoter X-box regions, PCR bands were detected from samples immunoprecipitated with RFX4 antibodies (lower panel). Lane f/+: the PCR band was from control mouse DNA as a positive control; lane IgG: mouse IgG as a negative control; lane IP: RFX4 antibody was used to immunoprecipitate DNA. (C) Schematic diagram of the *Foxj1* X-box constructs, each representing where the putative X box is located on the genomic region. There are two putative X-boxes in the *Foxj1* proximal promoter region. The lowest illustrated construct contains no X-box and served as a negative control. (D) Luciferase reporter assay. The *Dync2li1* X-box sequence cloned into the luciferase reporter vector was *trans*-activated to the greatest extent by *Rfx4* cDNA overexpression in the cellular system (p< 0.0001), whereas there was relatively less *trans*-activation by X-boxes from *Foxj1* and *Ift172* promoters (p< 0.01). Fold changes were calculated by normalization to the expression of the pGL4.23 vector. The assay was carried out in triplicate, and the results were presented as fold change, with the values representing the means from three independent transfection experiments.

In previous studies, Zhang et al showed that RFX4 isoform 1 could bind directly to the X-box of the human C-X3-C Motif Chemokine Ligand 1 (*CX3CL1*) gene promoter and stimulate its expression [23]. Using a yeast two-hybrid screening approach, nine potential RFX4 isoform 1 interacting partners were identified, including the G-protein pathway suppressor 2 (GPS2) [24]. In the point mutation *Rfx4 ^L298P^* model, intra-flagellar protein 172 (*Ift172*) was identified as an RFX4-regulated gene by ChIP-seq sequencing [22]. In the current study, first we measured the expression of transcripts encoding several cilia-related genes by qPCR from control and *Rfx4*^-/-^ embryo brain extracts. We found that *Foxj1* transcripts were decreased significantly by approximately 4-fold in the *Rfx4*^-/-^ embryos (p< 0.001) (Fig 4A), but dynein cytoplasmic 2 light intermediate chain 1 (*Dync2li1*), *Ift172*, and thrombospondin 1 (*Thbs*) mRNAs remained unchanged. As expected, *Rfx4* transcripts were not detectable in the KO embryos (p<0.0001). We therefore attempted to test the direct binding of RFX4 isoform 1 to the *Foxj1* promoter using a ChIP assay.

The consensus sequence for RFX protein binding to DNA is 5′-GTNRCC(0–3 N)RGYAAC-3′ (where N is any nucleotide, R is a purine, Y is a pyrimidine, and the two half sites GTNRCC and RGYAAC are separated by 0–3 base pairs) [25]. This so-called X-box has been used as a DNA binding site in searches for RFX (1–5) activated genes in several studies. In order to test whether *Foxj1* was a direct target gene of RFX4 isoform 1, two pairs of primers were designed to produce two fragments of approximately 100 bp that spanned 2 putative X-box sequences in the *Foxj1* promoter. These two PCR fragments were enriched by immunoprecipitation of DNA with the RFX4 isoform 1 protein from lysates of whole mouse brain (Fig 4B). In order to confirm this result, a cDNA that expressed RFX4 isoform 1 was co-transfected with a luciferase reporter construct that contained sequences corresponding to the above PCR fragments (Fig 4C). Transactivation was observed by RFX4 isoform 1 expression in the NG 108–15 neuroblastoma cell line, after normalizing with an empty cDNA vector co-transfected with the same luciferase reporter (Fig 4D). Vectors containing each of the two putative *Foxj* 1 X boxes were *trans*-activated by RFX4 (p< 0.01); however, a vector containing both X box sequences was not activated to a greater extent than the vectors containing single X box sequences (Fig 4D). Vector Foxj1N contained the promoter sequence without an X box, and served as a negative control; this exhibited minimal luciferase activity (Fig 4D).

We found that *Dync2li1* and *Ift172* gene promoter fragments were also enriched by the ChIP assay, and PCR fragments were amplified that contained the putative X-box sequences in their respective promoters (Fig 4B lower panel). The fragment containing the *Dync2li1* X-box was *trans*-activated to a high level by RFX4 isoform 1 expression in the luciferase reporter system, (p< 0.0001) (Fig 4D), whereas relatively less *trans*-activation was seen with the *Ift172* promoter sequence (p< 0.01) (Fig 4D). Interestingly, *Foxj1* and *Dync2li1* have been reported to be RFX3 target genes as well [26], and another study showed that RFX4 could form heterodimers with RFX2 and 3 [27]. Our data suggested that these genes are also likely to be direct targets of RFX4 isoform 1. However, *Ift172* and *Dync2li1* mRNA levels remained unchanged in the *Rfx4* KO brains, possibly due to compensation of other *Rfx* gene family members in the brain, since *Rfx3* and *Rfx2* transcript levels were unchanged in the *Rfx4*^-/-^ embryos (Fig 4A).

## Discussion

We report here that heterozygous inactivation of *Rfx4* led to apparent hypoplasia of the SCO, and that acetylated tubulin-labeled cilia were detached, both of which could lead to non-communicating congenital hydrocephalus. Homozygous inactivation of *Rfx4* resulted in severe brain midline defects and failure of hemisphere formation, representing a form of holoprosencephaly, and demonstrated that RFX4 isoform 1 is crucial for early brain development. Although more detailed studies need to be performed, it is highly likely that RFX4 isoform 1 has a major function within ependymal cells. In support of this, preliminary data from *Rfx4^flox/+^* mice expressing a *GFAP*-Cre transgene, in which Cre recombinase expression was directed by the mouse glial fibrillary acidic protein (GFAP) promoter, targeting astrocytes and ependyma in the brain, revealed that they were also hydrocephalic. Further studies of these mice confirmed that *GFAP* Cre *Rfx4^flox/+^* mice exhibited hydrocephalus with SCO dysplasia [28]. Several earlier studies have demonstrated that *Rfx* genes can regulate the function of cilia in *C*. *elegans*, *D*. *melanogaster*, and mammals [29]. For example, the *Rfx* gene *daf-19* was shown to regulate ciliogenesis in *C*. *elegans* [30], and *Rfx* was also found to play a role in ciliated sensory neuron differentiation in *D*. *melanogaster* [31]. A large number of diseases have been linked to problems of cilia structure and function, and these have been collectively termed ciliopathies [32]. RFX1 controls *ALMS1* gene expression by binding to X-boxes [33], and mutation of *ALMS1* causes Alstrom syndrome, a primary ciliopathy [34]. RFX2 was found to control ciliogenesis in the zebrafish pronephros [35], as well as left-right patterning in *Xenopus* [36]. *Rfx3* KO mice exhibited abnormal cilia development in the nodal cilium [37], truncated cilia on immature β-cells of the pancreas [38], and hydrocephalus [9]. We have shown here that the mutant *Rfx4* mice represent another ciliopathy disease model. It has been demonstrated that cilia-related gene deletion of *Foxj1* (*Hfh4*), *Mdnah5* (dynein heavy chain), *Polaris* (*Ift188*), *Hydin*, *Spag6* (central pair-dynein adaptor), and *Tg737* (component of the intraflagellar transport particles) in mice all exhibited ependymal cilia structural and/or functional defects associated with hydrocephalus [39–43]. Although cilia defects are often associated with hydrocephalus, it is still not known whether the cilia defects are primary, or are secondary effects of the hydrocephalus. In contrast to motile cilia, primary cilia are thought to be non-motile. Primary cilia are present on almost every cell during vertebrate embryogenesis, where they play major roles in signal transduction. In the homozygous *Rfx4 ^L298P^* model, reduced Shh signaling with primary cilia defects in telencephalon and spinal cord were demonstrated [22]. Although we did not characterize primary cilia in the current studies, the *Rfx4*^-/-^ mice described here exhibited hypoplasia of the entire brain at E12.5, the forebrain formed a single structure at E14.5, and there was no bilateral hemisphere formation. This partially mimics human holoprosencephaly, in which there is often fusion of the lobes of the forebrain. To date, the most commonly observed genetic defect in holoprosencephaly in mice and humans is mutation in *Shh* [44]. It will be interesting to investigate whether *Rfx4* is an important target gene for Shh, and if deletion of *Rfx4* interrupts normal Shh response signaling pathways.

Overall, the three available mouse mutation models involving different mutations of the *Rfx4* gene all exhibited similar phenotypes, demonstrating RFX4’s important functions in normal brain and spinal cord development. One advantage of the current model is that can be used to create a specific gene deletion at various developmental times and cell types.

Phylogenetic X-box motif analysis has shown that X-box DNA footprints typically exist in ciliary gene promoters of RFX-expressing organisms within the unikonts [45]. RFX3, operating through X-boxes, was shown to regulate cilia-related genes in mice [26], including *Foxj1*, *Dync2li1*, *Dnahc5*, *Dnahc11*, *and Dnahc*. In the current experiments, we have demonstrated that *Foxj1* expression levels were significantly decreased in the KO embryos, and *Foxj1* was a direct target gene of RFX4 isoform 1 in transfection studies. The *Foxj1* promoter contains two putative X-box sequences, which were enriched by the RFX4 antibody. Furthermore, *Foxj1* expression can be *trans*-activated by *Rfx4* cDNA overexpression in cultured cells. FOXJ1 (formerly HFH-4) is a member of the forkhead box family of transcription factors [46], and it is expressed in ciliated cells of the respiratory, reproductive, and central nervous systems [15, 47–49]. FOXJ1 and RFX3 have been shown to co-regulate some common target genes [29]; in particular, they can cooperate to govern a specific motile ciliogenic program [26, 50, 51]. Our experiments indicate that both RFX4 and RFX3 proteins can *trans*-activate *Foxj1*, but apparently not synergistically (data not shown).

On the other hand, a recent study found that ectopic expression of Foxj1 in *Xenopus laevis* suppressed the expression of *Rfx4*, suggesting that *Rfx4* can be controlled by Foxj1 [52]. It will be interesting to test this direction of regulation in an *in vitro* model, which should increase our understanding of potential molecular crosstalk among regulators of primary and motile ciliogenesis. Since *Rfx4* KO embryos exhibited different phenotypes from those with *Rfx3* and *Foxj1* mutations, RFX4 appears to regulate different genes than RFX3.

It is worth mentioning that *Mushashi1*, a neural stem cell marker, was recently identified as an *Rfx4* target gene [53]. *Mushashi1* knock out mouse exhibited hydrocephalus, and mutations of *Mushashi1* in patients were associated with microcephaly [54]. It is possible that aberrant *Mushashi1* expression in the current *Rfx4* KO model might account for some aspects of the phenotype described here.

In conclusion, we have identified *Foxj1* as an RFX4-regulated gene. In addition, our conditional KO mice provide a novel animal model in which to investigate downstream genes regulated by the RFX4 isoform 1 transcription factor in specific cell types and during specific periods of brain development. These studies may eventually provide insights into the pathogenesis of obstructive hydrocephalus and holoprosencephaly in humans, both relatively common and disabling birth defects. These studies should also lead to greater understanding of fundamental aspects of brain development, when brain-specific transcription factors like RFX4 isoform 1 are turned on and regulate downstream effector genes.
